# Integrated Transcriptome Analysis Reveals mRNA–miRNA Pathway Crosstalk in Roman Laying Hens’ Immune Organs Induced by AFB1

**DOI:** 10.3390/toxins14110808

**Published:** 2022-11-19

**Authors:** Zhongxian Xu, Qian Liu, Xueqin Liu, Maosen Yang, Yuan Su, Tao Wang, Diyan Li, Feng Li

**Affiliations:** 1 Key Laboratory of Southwest China Wildlife Resources Conservation (Ministry of Education), China West Normal University, Nanchong 637009, China; 2 Farm Animal Genetic Resources Exploration and Innovation Key Laboratory of Sichuan Province, Sichuan Agricultural University, Chengdu 611130, China; 3 College of Animal Science, Shanxi Agricultural University, Taigu 030801, China

**Keywords:** aflatoxin B1, transcriptional regulatory network, immune organs, chicken

## Abstract

Aflatoxin B1 (AFB1) is a widely distributed contaminant in moldy corn, rice, soybean, and oil crops. Many studies have revealed its adverse effects, such as carcinogenicity, immunotoxicity, and hepatotoxicity, on the health of humans and animals. To investigate the immunotoxic effects on chicken immune organs induced by AFB1, we integrated RNA and small-RNA sequencing data of the spleen and the bursa of Fabricius to elucidate the response of the differentially expressed transcriptional profiles and related pathways. AFB1 consumption negatively influenced egg quality, but no obvious organ damage was observed compared to that of the control group. We identified 3918 upregulated and 2415 downregulated genes in the spleen and 231 upregulated and 65 downregulated genes in the bursa of Fabricius. We confirmed that several core genes related to immune and metabolic pathways were activated by AFB1. Furthermore, 42 and 19 differentially expressed miRNAs were found in the spleen and the bursa of Fabricius, respectively. Differentially expressed genes and target genes of differentially expressed miRNAs were mainly associated with cancer progression and immune response. The predicted mRNA–miRNA pathway network illustrated the potential regulatory mechanisms. The present study identified the transcriptional profiles and revealed potential mRNA–miRNA pathway crosstalk. This genetic regulatory network will facilitate the understanding of the immunotoxicity mechanisms of chicken immune organs induced by high concentrations of AFB1.

## 1. Introduction

Aflatoxins are a class of secondary fungal metabolites, of which aflatoxin B1 (AFB1), a toxic compound, is the most common. The adverse reactions induced by AFB1, including acute toxicity, teratogenicity, mutagenicity, and carcinogenicity, have been well characterized in mammals and poultry [1,2]. For livestock, dietary AFB1 exposure can cause acute poisoning, lower production performance, reduced immunity, damaged organs, and even tumors and lethality, and the toxin residues in agricultural products ultimately endanger human health [3,4]. Several reviews have revealed the mechanism by which AFB1 induces cell apoptosis, inflammation, and DNA damage and repair [1,5]. As a classical model for immune-based studies that is functionally similar to humans, chickens have been used to test the activation and tolerance of aflatoxin-induced pathologies [6].

The bursa of Fabricius is a primary lymphoid organ specifically devoted to B-lymphocyte maturation and differentiation in the avian immune system [6,7]. The bursa is visible by embryonic day 5, and B-cell precursors rearrange *Ig* genes in the embryonic spleen prior to migration to the bursa beginning at the 8th embryonic day [8]. Subsequently, it develops with the maturation of B cells from the 15th day of incubation to the time of hatching, and B cells begin to emigrate to the peripheral lymphoid organs, such as the spleen, thymus, and harderian, to respond to antigens [9,10]. B-lymphocytes proliferate in the cortex and medulla util 8–10 weeks of age, when the bursa reaches its maximum size, and then a decrease in the number of lymphocytes in the interior of the medulla is accompanied by the involution of the bursa, which is completed by 6–7 months of age [11,12]. Jolly believes that the bursa grows rapidly up to the age of 4 months and then decreases in size [13,14], and Schauder indicated that the maximum size of the bursa is attained at 4–5 months of age [15]. Bruce proposed that the rate of bursa growth and regression differ between males and females, with female (12 weeks of age) bursa atrophy beginning 2 weeks later than that of males (10 weeks of age) in barred Plymouth rock × dominant white rock crosses. He also provided evidence that the atrophic changes in the bursa of Fabricius are largely variable among breeds, with the larger bursa presenting more rapid early growth in white leghorn chickens assumed to be a primary factor in their greater resistance to *Salmonella pullorum* [16]. Although the involution of the bursa is initiated at the age of approximately 8 weeks in white leghorn chickens, scattered atrophic or cystic follicles emerge at 20 weeks, become obvious at 24 weeks, and are essentially complete by 26 weeks, and cicatrized vestiges of bursa are visible at 28 weeks of age [10].

In poultry, AFB1 can induce lymphocyte reduction in the thymus, lymphoid follicle atrophy, and apoptotic cell increases in the thymus and the bursa of Fabricius [17], as well as elevating the expression levels of the apoptosis-related genes *Bax* and *Caspase*-3 in the thymus, while the expression level of *Bcl*-2 decreases with increasing AFB1 concentration [18,19]. AFB1-induced tissue changes develop with histopathological lesions, including visible congestion in the spleen and thymus and nuclear debris accumulation in the bursa [20,21]. In addition, AFB1 evokes a reduction in lymphocinesia and the depletion of lymphocytes in the bursa [20,22].

The spleen, a secondary lymphoid organ, is an important place for the proliferation and immune response of immunocompetent cells and the production of antibodies and effector cells. B cells from the bursa of Fabricius and T cells from the thymus have strong phagocytosis and filtering effects [18]. The percentage of T-cell subsets in the spleen is an important index representing the composition of mature T cells in vivo, which determines the biological function of mature T cells and ultimately involves the body’s cellular immune function [21]. Peroxidative damage is caused by adverse dietary AFB1 in chicks, exhibiting lesions of the spleen, which presents with a lighter color, smaller size, lower antioxidant capacity, and more lipid peroxidation products [18]. Its histopathological changes include red pulp congestion, splenic sinus dilatation, and small focal eosinophilic infiltration [23]. Moreover, AFB1 induces mutations in splenic lymphocytes and reductions in CD4+ and CD8+ T-cell numbers in rats [24]. Studies have emphasized that wild turkeys possess a more efficient resistance capacity to AFB1 exposure than domesticated turkeys [25]. AFB1 can lead to an inflammatory response in the spleen; an increase in proinflammatory cytokines, including *IFNγ*, *IL6*, and *TNFα*, in serum; and even the ingestion of low-dose AFB1 by chickens [26].

Given the particular vulnerability of livestock to aflatoxin contamination, many attempts have been made to elucidate the adverse effects of AFB1 on animal performance and immunity [27,28,29]. The toxicological effects of AFB1 have been well characterized in broilers [30,31,32], and our previous study established the dose–effect relationship between AFB1 and hepatotoxicity in Roman laying hens. Highly toxic AFB1 upregulated the expression of *PPARG* and downregulated the expression of *Bcl-6*, together with the dysregulation of gga-miR-301b-3p, gga-miR-301a-3p, gga-miR-190a-3p, and 2 cis-regulated lncRNAs [33]. In this study, to better understand the genetic regulatory networks of lymphoid organs induced by high concentrations of AFB1, we collected the bursa of Fabricius and the spleen as representatives of the central and peripheral lymphoid organs, respectively. mRNA and miRNA profiles were constructed to explore the core regulators that participate in pathologies such as cancer and immune diseases. The identified potential candidate miRNA–mRNA regulatory pairs will facilitate the comprehension of the molecular mechanisms involved in the transcriptome response to the toxic effects on lymphoid organs induced by AFB1.

## 2. Results

### 2.1. Egg Quality Was Adversely Affected by Dietary AFB1

**Figure 1:**
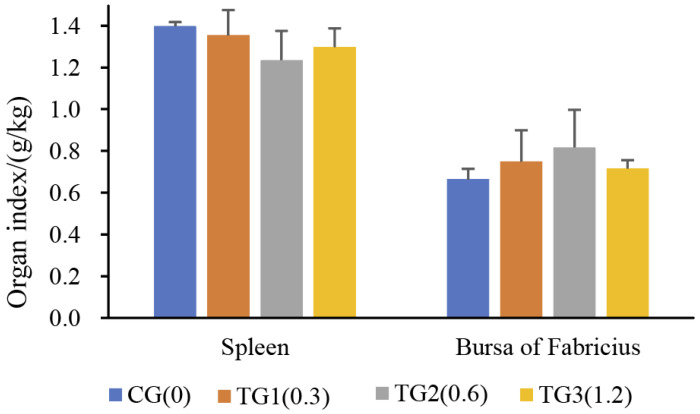
The effects of different concentrations of AFB1 on chicken organ indices. (CG: control group with 0.0 mg/kg AFB1; TG1, TG2, and TG3: groups treated with 0.3, 0.6, and 1.2 mg/kg AFB1, respectively).

Calculations were performed to determine the egg contents and eggshell indices to estimate the adverse effects of AFB1 on egg quality. No significant variation was observed in egg and eggshell weight. The yolk index and albumen height of the AFB1-treated chickens were significantly higher, while the eggshell quality was significantly reduced. Specifically, the eggshell thickness varied from 0.37 to 0.29 mm, and the breaking strength decreased from 4.49 to 3.72 kg (Table 1). These results indicated the weakened antibacterial efficiency and resistance to mechanical damage of the eggshells of birds treated with AFB1. The weight indices of the spleen and the bursa of Fabricius of the laying hens were not significantly (*p* > 0.05) influenced by different levels of AFB1 during the 5-week experimental period (Figure 1).

### 2.2. Transcriptional Profiles of the Chicken Immune Organs

We independently performed the transcriptional sequencing of the spleen and the bursa of Fabricius from the negative controls and the AFB1-treated groups (1.2 mg/kg). A total of 709.29 million raw reads were generated, and 106.39 Gb clean reads with an average depth of 8.87 Gb per library were obtained. Among these, 49.38 and 57.01 Gb clean reads were obtained, and 87.87% and 89.75% were mapped to the reference genome (Gallus gallus 6.0) for the spleen and the bursa of Fabricius, respectively (Table 2).

Furthermore, we obtained 5.71 Gb of clean data by processing 158.56 Mb raw reads from six small RNA sequencing libraries, corresponding to 2.38 Gb from the spleen and 3.33 Gb from the bursa of Fabricius. More than 77% of these miRNAs were mappable and could be aligned to unique miRNAs (Table 3).

### 2.3. Transcriptional Response to AFB1 Consumption

The expression level of transcripts was quantified and normalized to the log_2_(FPKM + 1) value. There were 6333 significantly differentially expressed genes (DEGs), 3918 upregulated and 2415 downregulated genes, in the AFB1-treated spleens compared to the controls (Table S1). A total of 296 DEGs consisting of 231 upregulated and 65 downregulated genes were detected in the bursa of Fabricius, with the threshold of |log_2_(Fold_change)| > 1 and *p* < 0.05 (Table S2).

Nine known and one novel miRNA were found in the spleen, whereas eight known and two novel miRNAs were detected in the bursa of Fabricius. We found that the overwhelming majority of the 10 most differently expressed miRNAs were tumor-suppressive (Table 4). Nine highly expressed miRNAs were core miRNAs in immune-mediated tumors, exerting anti-inflammatory effects in poultry diseases (Table 4, references in bold).

**Figure 2:**
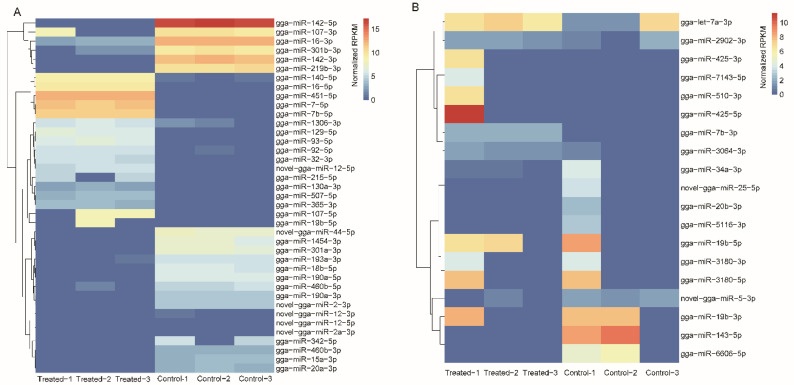
Heatmap of differentially expressed miRNAs in the spleen (**A**) and bursa of Fabricius (**B**).

Of the 780 detected miRNAs in the spleen, 673 were known and 107 were potentially novel miRNAs (Table S3). Of these miRNAs, 42 (22 upregulated and 20 downregulated) were identified from the spleen with the threshold of |log_2_(Fold_change)| > 1 and *p* < 0.005 (Figure 2A). In parallel, we identified 653 miRNAs, consisting of 593 known and 60 novel miRNAs, in the bursa of Fabricius (Table S4). Compared with the control library, nine upregulated and ten downregulated DEMs were screened (Figure 2B).

### 2.4. Activation of Immune- and Metabolism-Related Pathways after AFB1 Consumption

**Figure 3:**
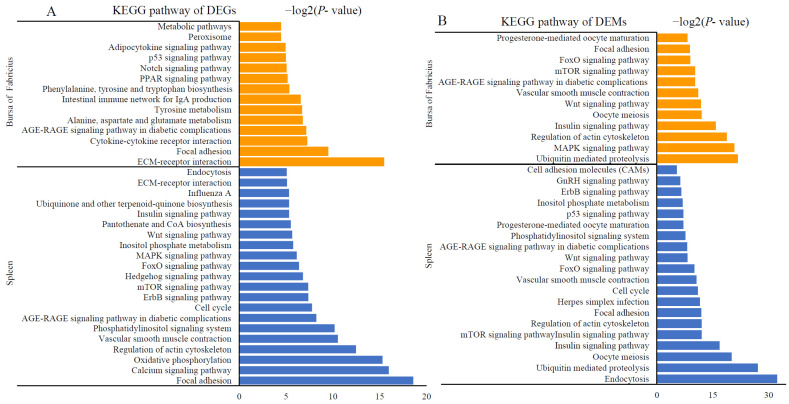
KEGG pathways significantly enriched in the differentially expressed mRNAs (**A**) and miRNAs (**B**) (orange and blue bars indicate pathways enriched in the spleen and the bursa of Fabricius, respectively).

We performed functional enrichment to elucidate the functions of the significantly differentially expressed mRNAs and miRNAs after AFB1 consumption. The KEGG pathways enriched by DEGs are illustrated in Figure 3A. For the spleen, the calcium signaling pathway, mTOR signaling pathway, and FoxO signaling pathway were associated with apoptosis. The ErbB signaling pathway, Hedgehog signaling pathway, and Wnt signaling pathway were related to carcinogenesis. Endocytosis, influenza A, and ECM–receptor interactions were identified as predominantly enriched immune response pathways. According to the functional enrichment of the bursa of Fabricius, metabolism, cytokine–cytokine receptor interaction, the p53 signaling pathway, the intestinal immune network for IgA production, and the PPAR signaling pathway were enriched by DEGs in the bursa of Fabricius. Based on the KEGG analysis of the target genes of DEMs in the spleen and the bursa of Fabricius, the main enriched pathways were consistent with those of the DEGs (Figure 3B).

**Figure 4:**
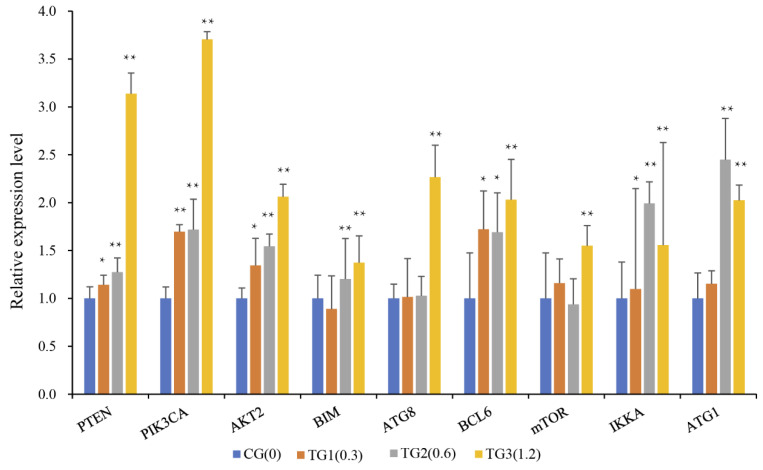
Validation of the genes involved in pathways with cancer progression and immune response (* indicates *p* < 0.05, ** indicates *p* < 0.01; CG: control group with 0.0 mg/kg AFB1; TG1, TG2, and TG3: groups treated with 0.3, 0.6, and 1.2 mg/kg AFB1, respectively).

We selected nine genes, *Akt2*, *PIK3CA*, *PTEN*, *BIM*, *ATG8*, *BCL6*, *MTOR*, *IKKA*, and *ATG1*, that were enriched by the pathways mentioned above and examined them independently by qPCR in chicken spleens. The results showed that a high concentration of AFB1 could significantly activate genes participating in cancer progression and immune response pathways (Figure 4).

### 2.5. Integrated Analysis of DEGs and DEMs

The potential target genes of DEMs were predicted by TargetScan and miRBase; target genes that were identified with a high level of confidence by both algorithms were retained, and target genes found to be DEGs were designated “intersecting genes” (Table S5). Of the 42 DEMs identified in the spleen, we screened 19 DEMs targeting 53 DEGs that were designated intersecting genes. Among these, 39 target DEGs were regulated by the 11 upregulated DEMs in the AFB1-treated group compared with the control group, and 37 DEGs were targeted by eight downregulated DEMs in the AFB1-treated group. As for the bursa of Fabricius, we identified two potential DEGs, *OTUD1* and *CPNE2,* that could be regulated by gga-miR-19b-5p and gga-let-7a-3p, respectively. Specifically, gga-miR-19b-5p was a DEM in both immune organs, as well as the liver in our previous study. Furthermore, we found 41 out of 105 DEGs were predicted as target genes of six highly expressed DEMs (gga-miR-16-5p, gga-miR-32-5p, gga-miR-301a-3p, gga-miR-142-3p, gga-miR-142-5p, and gga-miR-19b-5p), which suggested that these miRNAs might have important functions in disease and anti-inflammation by regulating their target genes.

### 2.6. Crosstalk between miRNA–mRNA and Pathways in Cancer Progression and Immune Response

**Figure 5:**
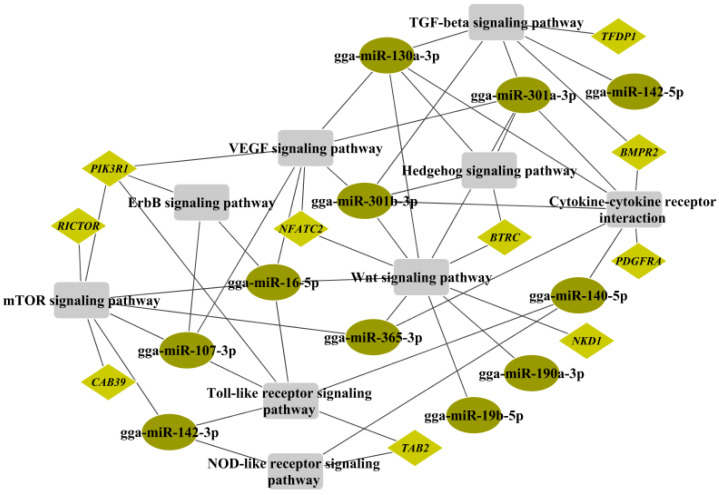
Regulatory networks between miRNA–mRNA pairs and pathways associated with cancer progression and immune response (the ellipses, diamonds, and rectangles indicate mRNA, miRNA, and pathways, respectively).

We found that the Wnt signaling pathway, VEGF signaling pathway, mTOR signaling pathway, ErbB signaling pathway, TGF-beta signaling pathway, and Hedgehog signaling pathway were associated with cancer. Functional genes, such as *PLCB4*, *NKD1*, *NFATC2*, *BTRC*, *PIK3R1*, *RICTOR*, *CAB39*, *TFDP1,* and *BMPR21*, corresponding to 10 regulated miRNAs (gga-miR-19b-5p, gga-miR-365-3p, gga-miR-190a-3p, gga-miR-16-5p, gga-miR-301a-3p, gga-miR-301b-3p, gga-miR-130a-3p, gga-miR-107-3p, gga-miR-142-3p, and gga-miR-142-5p) were found to participate in these pathways and play important roles in the process of tumor formation. In addition, the NOD-like receptor signaling pathway, Toll-like receptor signaling pathway, and cytokine–cytokine receptor interactions were significantly enriched and related to the inflammatory response. *PIK3R1*, *TAB2*, *PDGFRA*, and *BMPR2* were the main functional genes involved and were targeted by eight DEMs (gga-miR-107-3p, gga-miR-16-5p, gga-miR-142-3p, gga-miR-140-5p, gga-miR-365-3p, gga-miR-301a-3p, gga-miR-301b-3p, and gga-miR-130a-3p). The regulatory network of the mRNA–miRNA pathway with a high correlation is illustrated in Figure 5.

## 3. Discussion

Mycotoxins are considered the most accessible natural contaminants in animal diets, and recent studies suggest that both the level and length of AFB1 exposure affect the level of weight gain reduction. Although the regression of the bursa of Fabricius was found to be parallel with the development of the gonads [9], we observed the bursa of Fabricius in our study. In white leghorn chickens, a larger bursa with a higher early growth rate is associated with greater resistance to micro-organisms [16], and the involution of the bursa begins at the age of approximately 8 weeks and is essentially completed by 26 weeks [10]. Additionally, the growth and regression of the bursa is partially dependent on body weight increase [16]. The bursa of Roman laying hens was believed to regress at a much later age. Furthermore, the bursa plays a prominent role in antibody production and in the defensive mechanisms of the body [47]. Bruce suggested that the prolonging of bursa growth might be the result of external stress factors that stimulate the bursa to extend its growth and continue in its role as a defensive organ [16]. Therefore, AFB1 attack, as a canonical external stress factor, might be a prominent cause of stimulation to the bursa in peak laying hens.

During the experimental period, feeding with different AFB1 diets for 35 days had no apparent effect on the weight of eggs and the immune organ (the spleen and the bursa of Fabricius) indeces. The AFB1 diet negatively influenced eggshell quality, suggesting the weakened antibacterial efficiency and resistance to mechanical damage of shells treated with AFB1. There is evidence that a large immune organ index indicates a well-developed organ with strong immune function [48]. The spleen contains abundant lymphocytes, and its size can reflect the immune status of the body. The severe depletion of lymphoid cells and the rapid atrophy of the bursa of Fabricius were caused by NDV infection [49]. The variation trend of immune organ weight gain or loss in our study was consistent with previous reviews [3], indicating reduced immune capacity. However, there were no statistically significant differences between the AFB1-treated group and the control group, which might be due to the small number of subjects. It therefore seems that immune organs may adapt to an on-going dietary AFB1 challenge, and older chickens (165 days of age) exposed to AFB1 for a long time (5 weeks) might have enhanced adaptive immune tolerance and response. In summary, hens receiving AFB1 for 5 weeks showed a reduction in egg quality and adverse effects on immune organs.

There was evidence that the effect of a low dose (<1.0 mg/kg) of AFB1 on chicken performance was not consistent with its generalization [3]. To eliminate the inconsistency of the low dose effect, we chose 1.2 mg/kg AFB1-treated laying hens to explore the transcriptomic changes and elucidate the mechanism of AFB1-induced immunotoxicity and genotoxicity. Among the 20 most highly differently expressed miRNAs, nine were thought to be involved in poultry diseases. For instance, gga-miR-451 was demonstrated to suppress Newcastle disease virus (NDV)- and *Mycoplasma gallisepticum* (*MG*)-induced inflammatory responses by targeting YWHAZ [34,35]. gga-miR-16-5p was upregulated in infectious bursal disease virus (IBDV)- and *M. gallisepticum* (MG)-infected chickens, and it enhanced infectious apoptosis by targeting *Bcl-2* to exert an anti-inflammatory effect [37,38]. gga-miR-219b had a suppressive effect on tumour cells by targeting *BCL11B* [50]. The inhibitory effects of gga-miR-142-3p and gga-miR-142-5p on poultry disease tumorigenesis were revealed in [41,42]. Although there have been no studies on the inflammatory response of gga-miR-19b-5p in chicken diseases, Chen et al. have proven that gga-miR-19b-3p activates NF-κB signaling in host defense against Newcastle disease virus infection by targeting *ZMYND11* and *RNF11* [44]. Furthermore, gga-miR-7b-5p and gga-miR-1454-3p were found to be highly expressed in virulent infectious kidneys and were inferred to be associated with the pathogenesis of infectious bronchitis virus (IBV) [36]. gga-miR-6606-5p might be involved in the regulation of T, B lymphocyte activation as an immune response by targeting the gene *BLM*, which is localized in the QTL region and related to the spleen index [51]. In our previous study, miR-301a-3p in chicken liver was found to participate in these pathways, which might play important roles in the process of tumor formation [33]. Furthermore, in humans, miR-32-5p [39,40], miR-425-5p, miR-425-3p [43], miR-3180-5p [45], and miR-143-5p [52] are widely studied miRNAs in clinical practice, and they might be reliable prognostic and predictive tools for disease recurrence in various cancer patients.

The mRNA–miRNA pathway regulatory network showed that six pathways were cancer-related and corresponded to ten DEMs, and another eight DEMs were involved in three inflammatory response pathways by targeting four DEGs, hinting that AFB1 could trigger an imbalance of physiological signaling pathways via the mediation of miRNA–mRNA regulatory pairs. This finding was consistent with previous studies that identified the diseases induced by AFB1 [53,54]. For instance, a set of chicken cytokines/chemokines including IL-4, IL-6, TGFβ, and IFNs are upregulated in vvIBDV (very virulent infectious bursal disease virus)-infected spleens and the bursa of Fabricius [55]. The NOD-like receptor signaling pathway, the cytokine–cytokine receptor interaction signaling pathway, and the Toll-like receptor signaling pathway were significantly enriched in the bursa of Fabricius in chickens during infection with vvIBDV [56]. Furthermore, the cytokine–cytokine receptor interaction signaling pathway and the Toll-like receptor signaling pathway were enriched in the NDV-infected bursa of Fabricius in chickens [57]. Toll-like receptors (TLRs) are expressed on antigen-presenting cells and play an important role in sensing pathogenic agents and inducing adaptive immunity. TLRs were upregulated in *E. coli*-infected chicken spleens and the bursa of Fabricius, enhancing the inflammatory response [58,59]. TAB2, an activator TGF-β-activated kinase 1, is required for numerous stimuli, such as TNFα, IL-1β, and TLR ligands, to induce the activation of NF-κB and MAPKs [46]. Gga-miR-16-5p and gga-miR-142-3p exerted inhibitory effects on poultry disease tumorigenesis [37,38,41,42]. It was postulated that gga-miR-107-3p, gga-miR-140-5p, *TAB2,* and *PIK3R1* might be associated with the immune mechanism against AFB1 challenge.

In the Wnt signaling pathway network, our previous study revealed that gga-miR-301b-3p, gga-miR-190a-3p, and gga-miR-365-3p played important roles in the process of apoptosis in AFB1-treated chicken livers [33]. gga-miR-16-5p is reported to be dysregulated in infected chickens and correlated with thymic immunity through cytokine–cytokine receptor interactions and the Jak-START signaling pathway [60], and this miRNA inactivated the PI3K/Akt/NF-κB pathway by directly affecting its target gene PIK3R1 [38]. gga-miR-19b-3p activates NF-κB signaling in host defense against NDV in chickens [44]. NFATC2 is a transcription factor expressed in most immune system cells that plays a pivotal role in T and B cell activation and immune response [61]. The expression of IL-4 and IL-5 was distinctly enhanced in the lymph node and spleen cells of infected NFATC2-deficient mice [62]. Many proinflammatory cytokines, including TGF-β, were downregulated, and the pathways involved in the coagulation system, prothrombin activation, and acute-phase response were significantly altered in AFB1-infected domesticated turkeys [63].

Furthermore, several cancer-related pathways were significantly enriched in AFB1-infected chicken immune organs, such as the mTOR signaling pathway, VEGF signaling pathway, ErbB signaling pathway, and Toll-like receptor signaling pathway, which was consistent with previous studies. Several reviews have revealed the involvement of AFB1 in cancer development [1,19], and a set of genes/miRNAs that can be used as targets to evaluate the damage induced by AFB1 and its capacity to induce cancer has been identified. These genes are involved in the PI3K-Akt signaling pathway, MAPK signaling pathway, TNF signaling pathway, and other pathways mentioned in this study [19].

## 4. Conclusions

The consumption of a high concentration of AFB1 negatively influenced egg quality and immune organs. AFB1 challenge can activate core genes related to immune and metabolic pathways; the majority of the most high differentially expressed miRNAs were associated with poultry diseases. A series of genes and miRNAs associated with cancer progression and immune response were identified by predicting the mRNA–miRNA pathway interaction network. This phenomenon probably leads to an inflammatory response, an increase in T and C cell numbers, and the reduced production of cytokines by T cells in the spleen and the bursa of Fabricius. The inferred regulatory relationships between miRNAs and target genes involved in the immune mechanism responsible for resistance to AFB1 toxicity need further confirmation.

## 5. Materials and Methods

### 5.1. Animals, Diets, and Sampling

Chickens (Roman laying hens) were raised in the Avian Farm of Sichuan Agricultural University (Ya’an, Sichuan Province, China). A total of 24 healthy adult hens (1.4 ± 0.2 kg) at the age of 165 days were randomly divided into 4 groups, each containing 6 hens; the members of the groups were supplemented with 0.0 mg/kg (control group: CG), 0.3 mg/kg (treated group 1: TG1), 0.6 mg/kg (treated group 2: TG2), and 1.2 mg/kg (treated group 3: TG3) dietary AFB1, respectively. The AFB1-containing diet was prepared as follows: AFB1 (Sangon, Shanghai, China) was dissolved in DMSO and mixed with the basic diet, and the negative control diet was treated with an equal amount of DMSO. The nutritional contents of the basic diets were adequate according to the National Research Council (NRC) (1994) standards [64]. All hens were kept in cages and provided with ad libitum access to water and food throughout the 5-week experimental period. Eggs were collected every day to determine the effects of AFB1 on egg quality.

After 5 weeks of treatment, all the experimental hens were sacrificed to collect the spleen and the bursa of Fabricius, of which 6 replicates were used to detect the organ indices for the groups treated with a low level of AFB1 (TG1 and TG2). To avoid RNA degradation, 3 replicates were flash-frozen in liquid nitrogen and then stored at −80 °C until RNA extraction, and another 3 replicates were used to detect the organ indices for the control group (CG) and the group treated with a high level of AFB1 (TG3). All experiments were conducted in compliance with the guidelines approved by the Institutional Animal Care and Use Committee of Sichuan Agricultural University (DKY-S20160906), and all efforts were made to minimize animal suffering.

### 5.2. Measurements of Egg Quality and Organ Indices

The quantitative traits of the eggs were determined at weekly intervals by randomly testing 15 eggs from each group. Specifically, egg weight was measured with an electronic scale, and the shape of the egg (length/breadth) was measured with a Vernier calliper. Subsequently, the eggshell breaking strength was measured by an eggshell force gauge. The eggshell was broken, washed, dried, and then weighed. The egg yolk was separated from the albumen and weighed by an electronic scale. The height of the albumen was measured with a tripod micrometer. The eggshell thickness of the blunt end, middle, and sharp end was measured with an eggshell thickness gauge. The fat and connective tissues surrounding the spleen and the bursa of Fabricius were removed and weighed with an electronic scale. The collected spleen and the bursa of Fabricius were rinsed with phosphate-buffered solution and weighed after blotting with filter papers. The immune organ index was the percentage of net weight divided by body weight. The variance in egg quality traits and organ weight was determined by one-way ANOVA in R. Multiple comparisons were applied to conduct Duncan’s test for the AFB1-treated groups (0.3, 0.6, and 1.2 mg/kg) and the control group (0.0 mg/kg).

### 5.3. RNA Isolation and Sequencing

Total RNA was isolated from the tissues using TRIzol reagent (Invitrogen, Carlsbad, CA, USA) following the manufacturer’s instructions. The quantity and purity of total RNA were monitored by a NanoDrop 1000 (NanoDrop, Thermo Scientific, Waltham, MA, USA) and 1% formaldehyde agarose gel electrophoresis. Both library construction and sequencing were performed on the Illumina HiSeq platform (ANNOROAD, Beijing, China).

### 5.4. Transcriptomic Data Analysis

The quality control of raw mRNA-seq reads was conducted using FastQC (v0.11.5), and clean reads were obtained after the removal of low-quality reads, adaptor sequences, and poly-N. Clean reads were mapped against the chicken reference genome (Gallus gallus 6.0) using HiSAT2.0 with default parameters. Mapped reads were assembled and merged into a transcriptome with String Tie v1.3.3 and a custom Python script. The FPKM (fragments per kilobase million) values were calculated to evaluate the gene expression level. The differentially expressed genes (DEGs) were identified by DESeq2 software with a threshold of Benjamini-adjusted *p* < 0.05 and |log_2_ (FC)| > 1.

Low-quality small RNA-seq raw reads (>20% bases with a mass value < 30) were filtered, and high-quality reads were obtained after removing adaptors and fragments (<18 nt and >30 nt in length), eliminating tRNA, rRNA, snRNA, snoRNA, and other noncoding RNAs. Known and novel miRNAs were identified using miRdeep2. The expression level of miRNA was normalized by RPKM (reads per kilobase per million mapped reads) value. miRNAs with |log_2_ (FC)| > 1 and FDR < 0.05 were considered differentially expressed miRNAs (DEMs) through edgeR software.

### 5.5. mRNA–miRNA Target Association Analysis

The targets of DEMs were predicted by the intersection of miRDB and TargetScan, and miRNAs with target genes matching DEGs were retained to explore the targeted regulatory relationship with mRNA, whose |log_2_ (FC)| > 1 values denoted them as putative targeted regulatory mRNA–miRNA pairs (all RNAs were differentially expressed). Metascape (http://metascape.org/gp/index.html, accessed on 10 January 2021) was used to determine the functional enrichment of intersected genes [65,66]. The visualization of the co-expressed network between mRNA–miRNA pairs and related pathways was performed by Cytoscape v3.6.1 [67].

### 5.6. Quantitative Real-Time PCR of Genes Associated with Interacting Pathways

The expression levels of genes were quantitated by performing qRT-PCR assays. Five micrograms of total RNA were used to synthesize cDNA using EasyScript One-Step gDNA Removal and cDNA Synthesis SuperMix (TransGen, Beijing, China) following the manufacturer’s protocol. Then, 100–150 ng cDNA was added as a template to conduct qRT-PCR amplification using TransStart Top Green qRT-PCR SuperMix (TransGen, Beijing, China). The primer sequences are listed in Table S6. Three replicates were used to conduct the experiment, and the amplified products were tested with 1.5% agarose gels and statistically analyzed by the comparative 2^−∆∆CT^ method.

## Figures and Tables

**Table 1:** The effect of different concentrations of AFB1 on chicken egg quality.

Variables	CG (0)	TG1 (0.3)	TG2 (0.6)	TG3 (1.2)
Egg weight (g)	52.37 ± 2.46	53.16 ± 2.40	52.6 ± 2.99	52.45 ± 3.81
Yolk percentage	0.26 ± 0.01 ^c^	0.27 ± 0.02 ^a^	0.26 ± 0.02 ^b^	0.28 ± 0.02 ^a^
Albumen percentage	0.61 ± 0.02 ^b^	0.61 ± 0.02 ^a^	0.61 ± 0.02 ^a^	0.60 ± 0.02 ^a^
Albumen height (mm)	5.65 ± 1.03 ^b^	5.90 ± 0.91 ^b^	5.26 ± 1.06 ^b^	6.09 ± 0.74 ^ac^
Eggshell percentage	6.56 ± 0.70	6.53 ± 0.68	6.66 ± 0.59	6.47 ± 0.46
Shell thickness of blunt end (mm)	0.37 ± 0.05	0.37 ± 0.04	0.37 ± 0.03	0.36 ± 0.04
Shell thickness of equator (mm)	0.37 ± 0.04 ^a^	0.36 ± 0.04 ^a^	0.36 ± 0.03 ^ab^	0.29 ± 0.15 ^b^
Shell thickness of sharp end (mm)	0.36 ± 0.06	0.38 ± 0.04	0.38 ± 0.04	0.38 ± 0.02
Shell strength (kg)	4.49 ± 0.80 ^b^	6.13 ± 9.23 ^a^	6.64 ± 11.61 ^ab^	3.72 ± 0.95 ^ab^
Shape index	1.30 ± 0.03 ^a^	1.29 ± 0.03 ^b^	1.29 ± 0.04 ^b^	1.26 ± 0.03 ^c^
Egg length (cm)	54.01 ± 1.12 ^a^	53.76 ± 1.22 ^b^	53.79 ± 1.34 ^b^	52.4 ± 1.36 ^c^
Egg width (cm)	41.45 ± 0.74	41.83 ± 0.59	41.59 ± 0.95	41.5 ± 1.43

Note: Data are shown as means ± SEM. Means with different superscript (a, b, c) in the same line for the same item differ significantly (*p* < 0.05). a, b and c represent the significant difference between the group and control group, TB1(0.3) TB2(0.6), TB3(1.2), respectively.

**Table 2:** Overview of mRNA sequencing data.

Tissue	Sample	Raw Reads (Mb)	Clean Reads (Mb)	Effective Rate (%)	Clean Reads (Gb)	Raw Reads (Gb)	Map Rate (%)
Spleen	TG-3	60.67	53.60	88.35	9.10	8.04	87.84
	TG-2	48.00	42.87	89.31	7.20	6.43	88.24
	TG-1	55.13	53.63	97.27	8.27	8.04	86.74
	CG-3	59.44	57.26	96.33	8.92	8.39	88.43
	CG-2	50.75	50.15	98.82	7.61	7.47	88.23
	CG-1	55.22	54.56	98.80	8.28	8.13	87.71
Bursa of Fabricius	TG-3	65.22	64.55	98.97	9.78	9.62	90.10
	TG-2	58.05	57.38	98.85	8.71	8.55	87.50
	TG-1	61.71	61.11	99.03	9.26	9.10	90.02
	CG-3	63.54	62.84	98.90	9.53	9.36	91.25
	CG-2	63.15	62.36	98.75	9.47	9.29	89.88
	CG-1	68.41	67.70	98.96	10.26	10.09	90.07

Note: TG and CG represent AFB1 dietary group at a concentration of 1.2 mg/kg and the negative control group (0.0 mg/kg), respectively.

**Table 3:** Overview of small RNA sequencing data.

Tissue	Sample	Raw Reads (Mb)	Clean Reads (Mb)	Effective Rate (%)	Raw Base (Gb)	Clean Base (Gb)	Map Rate (%)
Spleen	TG-3	12.75	10.71	84.03	0.64	0.54	74.70
	TG-2	15.53	12.86	82.78	0.78	0.64	76.00
	TG-1	10.55	8.49	80.46	0.53	0.42	74.80
	CG-3	12.84	11.21	87.29	0.64	0.25	82.80
	CG-2	14.12	12.53	88.75	0.71	0.28	78.60
	CG-1	13.25	11.56	87.24	0.66	0.25	81.00
Bursa of Fabricius	TG-3	16.27	13.52	83.10	0.81	0.68	69.30
	TG-2	9.46	7.72	81.60	0.47	0.39	73.70
	TG-1	8.82	7.42	84.07	0.44	0.37	74.90
	CG-3	16.29	13.75	84.40	0.81	0.69	70.70
	CG-2	14.52	11.74	80.88	0.73	0.59	75.00
	CG-1	14.16	12.11	85.52	0.71	0.61	74.30

Note: TG and CG represent AFB1 dietary group at a concentration of 1.2 mg/kg and the negative control group (0.0 mg/kg), respectively.

**Table 4:** The 10 most highly differentially expressed miRNAs.

	miRNA	LogFC	*p*-Value	References
Spleen	gga-miR-451-5p	−6.71	1.38 × 10^−13^	**[34,35]**
	gga-miR-7b-5p	−6.57	1.79 × 10^−12^	**[36]**
	gga-miR-16-5p	−6.30	1.39 × 10^−10^	**[37,38,39]**
	gga-miR-32-5p	−6.21	5.18 × 10^−10^	[39,40]
	gga-miR-1454-3p	5.76	1.78 × 10^−7^	**[36]**
	gga-miR-301a-3p	5.79	1.40 × 10^−7^	[33]
	novel-gga-miR-44-5p	5.87	5.79 × 10^−8^	-
	gga-miR-219b-3p	6.32	2.07 × 10^−10^	**[33]**
	gga-miR-142-3p	6.57	4.55 × 10^−12^	**[41,42]**
	gga-miR-142-5p	7.03	3.88 × 10^−16^	**[41,42]**
Bursa of Fabricius	gga-miR-425-5p	−4.97	6.46 × 10^−4^	[43]
	gga-miR-19b-5p	−4.65	3.57 × 10^−4^	**[44]**
	gga-miR-3180-5p	−4.43	4.95 × 10^−3^	[45]
	gga-miR-425-3p	−4.24	8.73 × 10^−3^	[46]
	gga-miR-510-3p	−4.20	9.92 × 10^−3^	-
	gga-miR-5116-3p	3.69	1.97 × 10^−2^	-
	novel-gga-miR-5-3p	3.70	1.92 × 10^−2^	-
	novel-gga-miR-25-5p	3.98	9.19 × 10^−3^	-
	gga-miR-143-5p	5.15	7.00 × 10^−5^	[40]
	gga-miR-6606-5p	5.40	1.34 × 10^−5^	**[41]**

Note: The references in bold, corresponding to nine highly expressed miRNAs, which were core miRNAs discovered in poultry diseases.

## Data Availability

The datasets generated for this study can be found in the National Center for Biotechnology Information (NCBI) with accession numbers PRJNA901530 (mRNA) and PRJNA901043 (sRNA).

## Supplementary Materials

The following supporting information can be downloaded at: https://www.mdpi.com/article/10.3390/toxins14110808/s1, Table S1: Differentially expressed genes of spleen; Table S2: Differentially expressed genes of bursa of Fabricius; Table S3: The TPM of miRNAs in spleen; Table S4: The TPM of miRNAs in bursa of Fabricius; Table S5: The targeting association between DEMs and DEGs in the spleen and bursa of Fabricius; Table S6: The primer sequences used for qRT-PCR.
